# Minimal important differences of EORTC QLQ-C30 for metastatic breast cancer patients: Results from a randomized clinical trial

**DOI:** 10.1007/s11136-021-03074-y

**Published:** 2022-01-04

**Authors:** Takuya Kawahara, Naruto Taira, Takeru Shiroiwa, Yasuhiro Hagiwara, Takashi Fukuda, Yukari Uemura, Hirofumi Mukai

**Affiliations:** 1 Clinical Research Promotion Center, The University of Tokyo Hospital, 7-3-1 Hongo, Bunkyo-ku, Tokyo, 113-8655 Japan; 2 Breast and Endocrine Surgery Department, Okayama University Hospital, Okayama, Japan; 3 Center for Outcomes Research and Economic Evaluation for Health, National Institute of Public Health, Wako, Japan; 4 Department of Biostatistics, Division of Health Sciences and Nursing, The University of Tokyo, Tokyo, Japan; 5 Department of Data Science, Center for Clinical Sciences, National Center for Global Health and Medicine, Tokyo, Japan; 6 Department of Breast and Medical Oncology, National Cancer Center Hospital East, Kashiwa, Japan

**Keywords:** Anchoring, EORTC QLQ-C30, Health-related quality of life, Minimal important difference, Patient-reported outcomes

## Abstract

**Purpose:**

To establish minimal important differences (MIDs) for the European Organisation for Research and Treatment for Cancer Quality of life Questionnaire core 30 (EORTC QLQ-C30) in patients with metastatic breast cancer.

**Methods:**

The dataset was obtained from the SELECT BC-CONFIRM randomized clinical trial. Anchors obtained from patients (transition items) and clinicians (performance status) were used for anchor-based methods. Anchors obtained through 6 months after starting treatment were used for this analysis. Correlation coefficients of anchor and change in QLQ-C30 and effect size were used to qualify for estimating MIDs. Mean change method and generalized estimating equation were applied to estimate MIDs. Distribution-based methods were used for comparison.

**Results:**

We analyzed a dataset of 154 metastatic breast cancer patients. MIDs were estimated in 8 of 15 scales of QLQ-C30. Estimated MIDs for within-group improvement varied from 7 to 15 and those for deterioration varied from − 7 to − 17. Estimated MIDs for between-group improvement varied from 5 to 11 and those for deterioration varied from − 5 to − 8 across QLQ-C30 scales. Patient-reported anchors were more susceptible to early changes in health status than clinician-reported anchors.

**Conclusion:**

We provided the MIDs of the QLQ-C30 using both patient- and clinicians-reported anchors measured in a randomized trial of Japanese patients with metastatic breast cancer. We recommend patient-reported anchors for anchor-based estimation of MID. Our results can aid patients and clinicians, as well as researchers, in the interpretation of QLQ-C30.

**Supplementary Information:**

The online version contains supplementary material available at 10.1007/s11136-021-03074-y.

## Introduction

Treatment for metastatic breast cancer aims to prolong survival and palliate symptoms [1]. Considering that metastatic breast cancer remains incurable, maintaining quality of life (QOL) is an important therapeutic goal [2]. Instruments to measure QOL in patients with metastatic breast cancer include the European Organisation for Research and Treatment of Cancer Quality of Life Questionnaire core 30 (EORTC QLQ-C30). Despite improvements in validation of the QLQ-C30, interpreting the numerical scores remains challenging.

A large clinical trial can show statistically significant differences for QOL scores, but the clinical relevance of these differences remains controversial. The concept of minimal important difference (MID) provides a measure of the smallest difference in QOL scores that patients can perceive as improvement or deterioration [3]. King has comprehensively reviewed the literature on this topic [4]. While both anchor- and distribution-based methods are available for estimating MIDs, the anchor-based method has the appeal of incorporating external instruments (criterion or anchor) that are relevant to patients [5].

Recommendations [6] or guidelines across cancer sites using meta-analysis [7, 8] have been provided to interpret QLQ-C30. These are useful for designing trials and interpretation, but MIDs can vary according to factors such as patient populations, cancer site, and disease stage. Research into MIDs for metastatic breast cancer remains limited. Musoro et al. recently used two published trials to estimate MIDs relying on clinical anchors from clinician examinations [9]. In contrast, the most widely used anchor is the rating of change in health status as obtained directly from patients [10].

The purpose of this study was to determine the MIDs of the QLQ-C30 in a population of metastatic breast cancer patients using data from the SELECT BC-CONFIRM trial. In that trial, transition items reported directly from patients and other clinical anchors were collected at multiple timepoints from the initiation of treatment; these trial data were thus considered desirable for the estimation of MID.

## Methods

### Patient population

The dataset for this study comes from the SELECT BC-CONFIRM randomized clinical trial, which enrolled and randomized 230 patients from multiple centers in Japan [11]. The trial aimed to confirm that S-1, an oral fluorouracil antitumor drug, is non-inferior to anthracycline-containing regimens as first-line chemotherapy for HER2-negative metastatic breast cancer. Patients were randomized to receive oral S-1 or intravenous anthracycline (doxorubicin/cyclophosphamide or epirubicin/cyclophosphamide) at a standard dose. The main inclusion criteria for the trial were as follows: presence of HER2-negative metastatic breast cancer; endocrine therapy-resistant status; and no previous administration of chemotherapy for advanced disease. The primary endpoint in that trial was overall survival, and QOL was included as a secondary endpoint. The research ethics boards of all participating institutions approved this study, and all participants provided written informed consent prior to enrollment. The trial was registered with the University Hospital Medical Information Network, Japan (UMIN000005449).

### The EORTC QLQ-C30

The EORTC QLQ-C30 is a general QOL instrument for cancer patients [12]. This questionnaire comprises 30 items, 24 of which are aggregated into five functional scales (physical, role, emotional, cognitive, and social), three symptom scales (fatigue, pain, and nausea/vomiting), and one global health status. The remaining six items assess additional symptoms (dyspnea, appetite loss, insomnia, constipation, and diarrhea) and financial impact. The SELECT BC-CONFIRM trial used the Japanese version of the QLQ-C30 questionnaire (version 3) [13], with each scale/item converted into a score ranging from 0 to 100. A high score in the functional scale represents increased functional ability, whereas a high score in the symptom scale represents increased distress. Researchers administered the QLQ-C30 before starting the protocol treatment and every 2 months thereafter until 1 year after starting treatment.

### Anchors

We selected two anchors: the patient’s global rating of transition; and change in Eastern Cooperative Oncology Group performance status (PS) [14]. The transition items comprised three questions: “Compared with when you started the chemotherapy in this trial: (a) How has the time you spend in bed or a chair during the day changed? (a question of function); (b) How have your concerns changed? (a question of concerns); and (c) How has your global health status changed? (a question of global health)”. We created the transition items for this study by referring to the item representativeness of each functional scale of the QLQ-C30 using factor analysis [15] of a previous clinical trial in Japan [16]. For each question, seven response options were provided, such as “much shorter”, “shorter”, “a little shorter”, “unchanged”, “a little longer”, “longer”, or “much longer” for the functional question. We devised the 7-grade responses based on the previous study [6]. In the trial, researchers administered the transition questionnaire with the QLQ-C30 every 2 months until 6 months after starting the protocol treatment. Researchers scored PS between 0 (fully active) to 4 (completely disabled) at every course of the treatment.

Three status groups were defined for transition items. At each timepoint, patients who made a response adjacent to “unchanged” were categorized as deterioration or improvement. Patients who responded two categories away from “unchanged” were excluded from analysis for estimation of MIDs, as these responses were considered beyond a “minimal” change. For example, patients who answered “a little longer”, “unchanged”, or “a little shorter” for the functional question were categorized as showing “deterioration”, “stable (no change)”, and “improvement”, respectively, and all other responses were excluded from the analysis of that timepoint. Similarly, at each timepoint, three change status groups were defined for the anchor of PS, calculated as the change in score from baseline: deterioration (worsened by one category of the anchor); stable (no change); or improvement (improved by one category of the anchor). Changes by two or more scores of PS were excluded from analysis, under the same rationale described above.

### Statistical analysis

We summarized patient characteristics and demographics of the analyzed cohort. The variables included age, weight, number of lymph node metastases, estrogen receptor status, progesterone receptor status, history of surgery, history of chemotherapy use, and history of oral fluorouracil use.

We compared changes in QLQ-C30 scores with anchors measured at 2, 4, and 6 months from baseline. We calculated correlation coefficient *r*, and considered *r* >|0.3| as necessary to show a moderate association between change scores and anchors [17]. We calculated Pearson’s correlation, Spearman’s rank correlation, polyserial correlation, and polychoric correlation. We compared the 0.3-threshold and the maximum of the four correlation coefficients to explore candidate scales of QLQ-C30.

We used the mean change method to estimate MIDs for within-group change. The within-group MID can be calculated in several ways; some scholars calculate mean change in a (slightly) improved/deteriorated group [18], while other scholars adjust the results by subtracting the mean change that occurs in the stable group [5, 9, 19, 20]. As the latter method has been more frequently applied in cancer research, we calculated the mean difference in change scores between deterioration and stable groups to indicate within-group MIDs for deterioration. Similarly, the mean difference of change scores between improvement and stable groups indicates MIDs for improvement. We calculated effect sizes (ESs) as the mean difference in change scores divided by the standard deviation (SD) of the QOL scale at baseline. ESs determine the responsiveness of changes in QOL to changes in anchor categories. We considered that ESs ≥ 0.2 and < 0.8 were appropriate to include as MIDs because an ES < 0.2 is small, and an ES ≥ 0.8 is large [21]. We calculated within-group MIDs separately for each pair of the anchor and timepoint (i.e., at 2, 4, and 6 months). To reduce the possibility of false-positive results, we show the results of QOL with at least an estimated two MIDs across timepoints or anchors.

We used a generalized estimating equation (GEE) to estimate MIDs for between-group change; we fitted the GEE for a given QOL and anchor pair, the changes in QOL over multiple timepoints as outcome variables, and anchor category as dummy explanatory variables. In contrast to the mean change method, the GEE method stacks changes in QOL scores over the timepoints and treats the scores as a vector of outcome. In comparative clinical trials in which patients respond to the QOL questionnaire longitudinally, the GEE with changes in QOL over multiple timepoints as outcome variables and treatment variable as explanatory variables is often fitted, rather than the simple linear regression which only uses a change in QOL at a specific timepoint. The GEE method thus provides a useful MID for between-group change [9]. We also applied the threshold for ESs (≥ 0.2 and < 0.8).

We obtained final estimates of MIDs provided by anchor-based methods using the correlation-based weighted average to reflect the degree of closeness of each pair of the anchor and change in QOL.

We also conducted a distribution-based method for MIDs. We calculated the 0.2 SD, 0.3 SD, 0.5 SD, and standard error of measurement based on a test–retest reliability of [22] for each QOL at all timepoints.

We conducted all statistical analyses using Base SAS and SAS/STAT version 9.4 software of the SAS System for Windows (SAS Institute, Cary, NC, USA).

## Results

In total, 230 patients were randomized in the trial. To calculate the change in QOL from baseline, we included only the 154 patients who had completed a baseline QLQ-C30. Table 1 summarizes the demographic and treatment characteristics of the analyzed cohort. Median age was about 60 years. The majority of patients had a history of surgery (76.6%), and about two-thirds of patients had no history of chemotherapy.

**Table 1:** Patient characteristics

	Anthracycline (*n* = 74)	S-1 (*n* = 80)	Total (*n* = 154)
Age (years), median (IQR)	60.5 (52–66)	59.5 (49–64)	60.0 (50–65)
Weight (kg), median (IQR)	52.0 (48–59)	53.0 (48–60)	52.0 (48–60)
Performance status			
0	61 (82.4)	64 (80.0)	125 (81.2)
1	13 (17.6)	16 (20.0)	29 (18.8)
Number of lymph node metastases			
0	24 (32.4)	20 (25.0)	44 (28.6)
1–3	17 (23.0)	27 (33.8)	44 (28.6)
4–9	11 (14.9)	6 (7.5)	17 (11.0)
> 9	5 (6.8)	7 (8.8)	12 (7.8)
Unknown	17 (23.0)	20 (25.0)	37 (24.0)
Estrogen receptor			
+	56 (75.7)	62 (77.5)	118 (76.6)
−	17 (23.0)	11 (13.8)	28 (18.2)
Unknown	1 (1.4)	7 (8.8)	8 (5.2)
Progesterone receptor			
+	45 (60.8)	52 (65.0)	97 (63.0)
−	26 (35.1)	21 (26.3)	47 (30.5)
Unknown	3 (4.1)	7 (8.8)	10 (6.5)
History of surgery			
No	18 (24.3)	18 (22.5)	36 (23.4)
Yes	56 (75.7)	62 (77.5)	118 (76.6)
History of chemotherapy			
No	49 (66.2)	57 (71.3)	106 (68.8)
Yes	25 (33.8)	23 (28.8)	48 (31.2)
History of oral fluorouracil			
No	63 (85.1)	69 (86.3)	132 (85.7)
Yes	11 (14.9)	11 (13.8)	22 (14.3)

Data represent *n* (%) unless stated otherwise. *IQR* inter quartile range

Numbers of patients by anchor categories and corresponding mean change in QOL scores are shown in Supplementary Table 1. When using the transition item of global health, the proportions of “improvement”, “stable (no change)”, and “deterioration” were 17%, 38%, and 23%, respectively, with the remaining 22% responding two or more categories away from “stable (no change)”. When using the change in PS anchor, the proportions of “improvement”, “stable (no change)”, and “deterioration” were 6%, 78%, and 13%, respectively. Table 2 shows correlation coefficients between change in QLQ-C30 and anchors. For each anchor, correlated QOL scales (i.e., maximum correlation coefficient > 0.3) at one or more timepoints are listed. The performance status showed a correlation with change in QLQ-C30, mostly at 6 months. Supplementary Table 2 shows correlation coefficients for absolute or baseline scores with anchors. In most pairs of anchors and scales, correlations of transition items with change scores were greater than correlations of transition items with absolute scores. In most pairs of positively (negatively) correlated transition items and scales, transition items were negatively (positively) correlated with the score at baseline.

**Table 2:** Correlations between changes in quality of life scales and anchors at 2, 4, 6 months from randomization

Anchor	Scale	2 months (*n* = 125–130)	4 months (*n* = 116–121)	6 months (*n* = 103–111)
Function	QL	**0.69**	**0.40**	**0.36**
	PF	**0.40**	**0.42**	**0.44**
	RF	**0.31**	0.29	**0.44**
	EF	0.17	**0.34**	**0.34**
	SF	**0.31**	**0.36**	0.27
	FA	**0.34**	**0.49**	**0.34**
	PA	0.15	**0.34**	0.26
	DY	**0.35**	0.16	0.15
	SL	0.07	0.25	**0.41**
	AP	**0.39**	0.24	0.17
Concern	QL	**0.33**	**0.36**	**0.30**
	PF	0.18	0.26	**0.34**
	RF	0.05	0.14	**0.36**
	EF	0.27	0.27	**0.46**
	SF	0.21	**0.40**	**0.34**
	PA	0.22	**0.31**	0.23
Global health	QL	**0.46**	**0.33**	**0.44**
	PF	**0.39**	**0.31**	**0.41**
	RF	**0.30**	0.27	**0.45**
	EF	0.22	0.23	**0.44**
	SF	**0.37**	0.27	**0.36**
	FA	**0.37**	0.24	**0.33**
	NV	**0.31**	0.04	0.18
	PA	**0.35**	**0.31**	**0.37**
	DY	**0.33**	0.08	0.19
	SL	0.06	0.12	**0.33**
	AP	**0.37**	0.21	**0.34**
Performance status	QL	0.14	**0.38**	0.25
	PF	0.17	0.25	**0.31**
	RF	0.20	0.17	**0.30**
	CO	0.03	0.21	**0.38**
	DI	0.08	0.16	**0.30**

Because the number of patients varied by quality of life scale and anchor, *n* is presented as a range for all scales and anchors. Correlations are shown in absolute values, with correlations >|0.3| shown in bold

*QL* global quality of life; *PF* physical function; *RF* role function; *EF* emotional function; *SF* social function; *FA* fatigue; *NV* nausea and vomiting; *PA* pain; *DY* dyspnea; *SL* insomnia; *AP* appetite loss; *CO* constipation; *DI* diarrhea

**Fig. 1:**
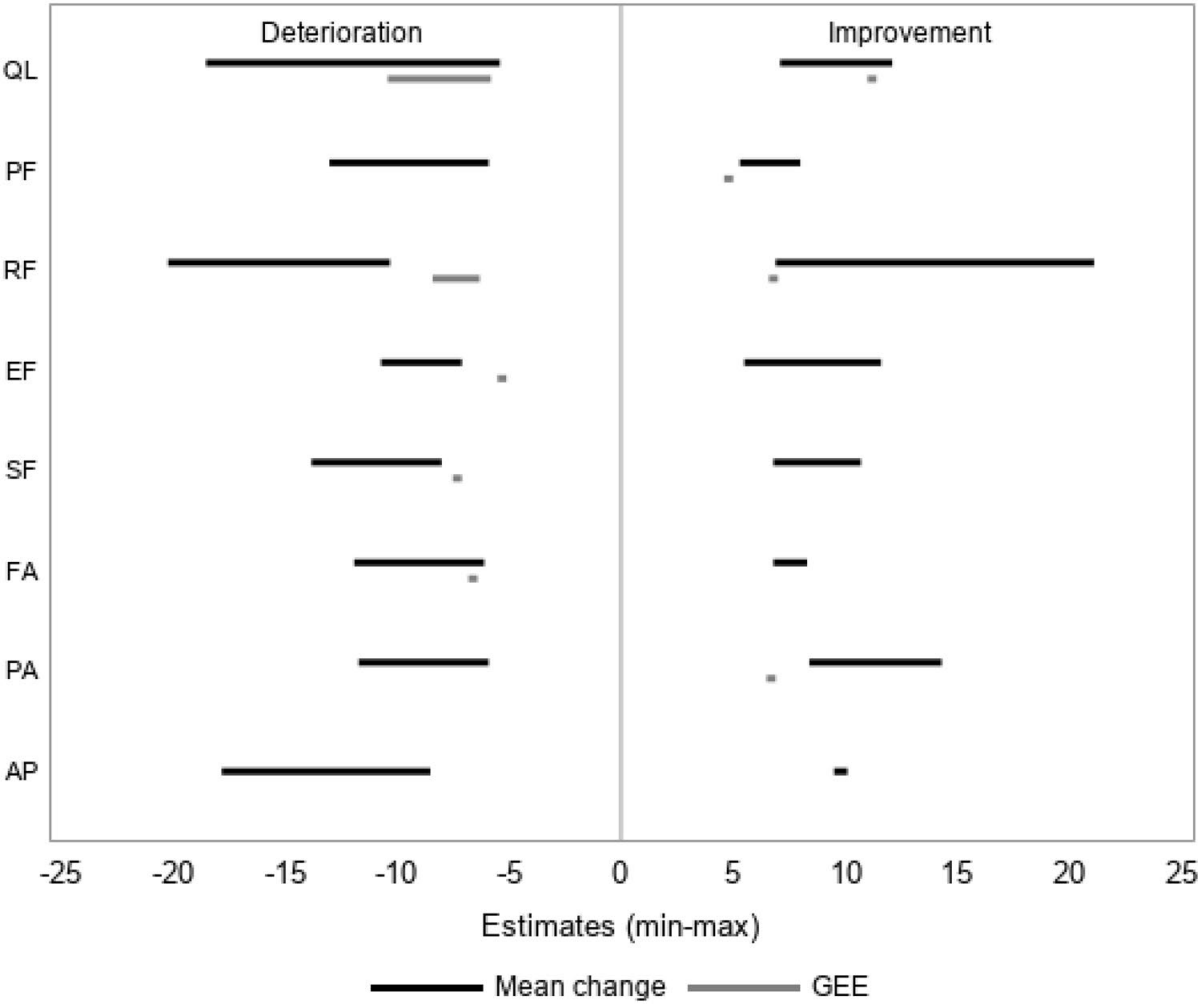
Minimal important differences obtained using the mean change method and generalized estimating equations. Symptom scales were reversed to align with functional scales; therefore, positive values represent decreases in symptom scales. Absence of the line (e.g., GEE estimates for deterioration in PF) indicates that no minimal important difference estimate that met our criteria was obtained. *GEE* generalized estimating equation; *QL* global quality of life; *PF* physical function; *RF* role function; *EF* emotional function; *SF* social function; *FA* fatigue; *PA* pain; *AP* appetite loss

Figure 1 shows the range of MIDs from the mean change method for each QOL scale across multiple anchors. The mean change method identified MIDs for improvement and deterioration in 8 of 15 QLQ-C30 scales, and the GEE identified MIDs for at least one direction in 7 of the 15 QLQ-C30 scales. Estimated MIDs differed between scales of QOL, direction (improvement and deterioration), and methods of estimation. Estimates from GEE tended to be smaller than those from the mean change method. No MIDs were estimated from GEE on some scales. Supplementary Table 3 shows details of results from Fig. 1. Most MIDs estimated with GEE were *p* < 0.05, but this was not the case for MIDs estimated under the mean change method.

Table 3 summarizes MIDs from anchor-based methods, alongside results from the distribution-based method for comparison. Distribution-based estimates at 2 months are presented in Table 3 because results at other timepoints were similar, with at most a difference of 0.8 units for 0.2 SD. Most MIDs for interpretation of within-group differences were between 0.3 and 0.5 SD.

**Table 3:** Summary of minimal important differences from anchor- and distribution-based methods

	Anchor-based method				Distribution-based method			
	Within-group		Between-group					
	Improvement	Deterioration	Improvement	Deterioration	0.2SD	0.3SD	0.5SD	SEM
QL	9	− 10	11	− 8	4.4	6.6	11.0	9.3
PF	7	− 9	5	No MID	3.7	5.6	9.3	5.6
RF	15	− 17	7	− 7	5.5	8.2	13.7	11.6
EF	9	− 7	No MID	− 5	4.0	6.0	10.0	7.5
SF	9	− 12	No MID	− 7	4.7	7.1	11.8	8.5
FA	7	− 10	No MID	− 7	4.3	6.5	10.8	8.9
PA	11	− 11	7	No MID	4.7	7.0	11.7	8.8
AP	10	− 9	No MID	No MID	5.9	8.8	14.7	13.5

Symptom scales were reversed to align with functional scales; therefore, positive values represent decreases in symptom scales

*SEM* standard error of measurement; *QL* global quality of life; *PF* physical function; *RF* role function; *EF* emotional function; *SF* social function; *FA* fatigue; *PA* pain; *AP* appetite loss; *MID* minimal important difference

## Discussion

To the best of our knowledge, this study is the first to estimate MIDs of QLQ-C30 in a population of Japanese patients with metastatic breast cancer. The study identified MIDs for interpreting within-group differences in 8 scales and for interpreting between-group differences in 7 scales.

As far as we know, only one study has examined the MIDs of QLQ-C30 in patients with advanced breast cancer [9]. Musoro et al. used two published trials to identify MIDs [9], and most of the patients were from European countries. They identified at least one suitable MID in eight scales, and six of those eight scales (global quality of life, physical function, role function, social function, fatigue, and appetite loss) were in common with the present findings. In these scales, MIDs for interpreting between-group were similar. Our results thus support their conclusions, at least for these scales.

Conversely, MIDs for interpreting within-group deterioration tended to be larger in our study than in the previous study [9], such as for global quality of life (− 10 vs. − 8), role function (− 17 vs. − 6), social function (− 12 vs. − 7), and fatigue (− 10 vs. − 8). Feng et al. [23] investigated systematic differences between Japanese and European general populations in self-reported QOL questionnaires. They found that the percentage of Japanese respondents reporting no problems in morbidity, usual activities, pain, or anxiety was higher than that of European respondents [23]. Our study of Japanese patients revealed that minimally deteriorated patients described larger changes from baseline than the European patients reported by Musoro et al. [9]. These findings may indicate inter-country differences in the self-evaluation of health at a single point as well as changes between two timepoints, especially in terms of deterioration.

Consistent with recent findings on MIDs for other cancer sites [5, 19, 24, 25], the magnitudes of MIDs for deterioration and improvement differed, along with the scales of QLQ-C30. We observed no systematic differences between previous results and our findings. These estimates generally do not conflict with the previously proposed guidelines [7, 8], although we consider that MIDs for interpreting deteriorations and improvements should be distinguished. In addition, MIDs for interpreting within- and between-group changes differed, as shown in Table 3. These results strongly indicate that MIDs fitted for purpose are necessary.

The choice of an anchor is one of the critical components in the estimation of anchor-based MIDs [10]. Musoro et al. [9] estimated MIDs relying on clinician-examined anchors such as performance status and common terminology criteria for adverse events. However, patient-reported anchors appear preferable to clinician-reported anchors [10]. The present study used both types of anchor: transition items and performance status. We found that transition items were more sensitive to early changes in QOL than performance status, as shown in Table 2. This finding is consistent with findings from previous research that self-reports from patients are more sensitive than clinicians’ reports to underlying changes in health status, and tend to identify clinically meaningful symptoms earlier [26, 27]. MID estimates by mean change methods using performance status as the anchor lay within the range of MID estimates using transition items as the anchor (Supplementary Table 3). This finding may indicate that clinician-reported anchors provide similar results to patient-reported anchors for estimating MIDs. However, we recommend patient-reported anchors as more susceptible to changes in the underlying health status of patients than clinician-reported anchors. Note that one needs to be cautious about the use of transition items because they are often vulnerable to recall bias.

Distinguishing between unreliable and credible MIDs is essential for MIDs to help patients and clinicians in making decisions. One such method is referring to a recent guideline [10] and reporting the credibility level of the anchors used to estimate MID. We created Supplementary Table 2 for this purpose. We note here two points. First, the correlations of transition items with change score were greater than those with absolute score. Otherwise, the transition item seems to capture current health status more than the change in health status. Second, transition items showed some correlation not only to the change in QOL, but also to QOL at baseline. This correlation reflects the fact that patients appropriately respond to transition items while considering the health status at baseline. These points support the credibility of the transition items used in this study.

Several limitations need to be considered when interpreting the present results. First, due to the small or medium sample size for estimating MID, each estimate was not precise. We aggregated estimates into a single MID using correlation-weighted averages, so we consider our final estimates as reliable to some extent. Second, no change group or improved or deteriorated group showed statistically significant results in some scales. We do not believe, however, that this seriously influenced our conclusions, because all estimates of MIDs in this study exceeded effect size thresholds.

In conclusion, we provided the MIDs of QLQ-C30 using both patient- and clinician-reported anchors measured in a randomized trial of Japanese patients with metastatic breast cancer. Our results can aid patients and clinicians, as well as researchers, with interpreting QLQ-C30. Further studies should replicate this study in other populations or other anchors to validate the current MID estimates.

## Supplementary Information

Below is the link to the electronic supplementary material.

Supplementary file1 (PDF 90 kb)

## Data Availability

The datasets generated and analyzed during the current study are available from the corresponding author on reasonable request.

## Code availability

The SAS codes generated during the current study are available from the corresponding author on reasonable request.
