# Supplementary material for: Minimal important differences of EORTC QLQ-C30 for metastatic breast cancer patients: Results from a randomized clinical trial
Source: Qual Life Res. 2022 Jan 4;31(6):1829–36. doi: 10.1007/s11136-021-03074-y (PMC9098551; doi:10.1007/s11136-021-03074-y)
Supplement: Supplementary file 1 — Supplementary file1 (PDF 90 kb) [file 11136_2021_3074_MOESM1_ESM.pdf]

Supplementary Table 1. The number of patients by anchor categories and corresponding mean change in QOL scores

Anchor	Scale	2 months			4 months			6 months		
		Imp	Stable	Det	Imp	Stable	Det	Imp	Stable	Det
Function										
N of patients		12	57–59	24–26	13	52–54	21–22	6–7	50–52	14–15
	QL	22.2	-0.3	-5.8	7.7	0.6	-1.9	4.8	5.8	-12.8
	PF	10.0	2.6	-5.6	10.0	2.2	-4.6	1.9	3.5	-9.6
	RF	6.9	0.0	-10.3	1.3	-3.1	-3.0	-2.4	1.3	-18.9
	EF	2.8	11.3	11.2	11.1	9.9	6.8	6.0	10.9	0.2
	CF	1.4	5.6	5.1	5.1	1.9	-0.8	-4.8	4.5	-11.1
	SF	2.8	3.4	5.1	9.0	2.2	0.8	-9.5	4.2	-11.1
	FA	-5.6	-0.9	5.1	-6.8	0.0	8.1	0.0	-6.0	5.9
	NV	-11.1	0.8	3.2	7.7	0.6	-4.5	16.7	-4.6	6.7
	PA	-8.3	-4.2	1.9	-1.3	-4.0	-0.8	0.0	-9.9	-3.3
	DY	-8.3	-3.4	5.1	0.0	-2.5	0.0	0.0	-5.8	11.1
	SL	-16.7	-10.3	-8.0	-10.3	-14.2	1.6	0.0	-7.7	15.6
	AP	-8.3	1.7	7.7	7.7	-0.6	1.5	9.5	-8.3	4.4
	CO	-5.6	3.4	5.1	-2.6	3.1	10.6	4.8	-0.6	6.7
	DI	11.1	5.6	3.8	10.3	5.6	3.2	4.8	5.2	-6.7
	FI	-13.9	0.0	-18.1	-20.5	-7.1	3.0	14.3	-2.7	-9.5
Concern										
N of patients		21–23	59–62	23	20–21	44–45	18–19	17	52–55	14
	QL	9.1	1.5	-4.0	8.7	0.6	-1.8	14.2	3.0	-6.0
	PF	-1.5	3.0	-0.3	5.1	1.6	-7.0	7.1	3.6	-5.5
	RF	-0.7	-1.1	-2.9	0.0	-1.9	-7.0	19.6	-1.5	-16.7
	EF	10.5	7.3	9.1	13.1	11.7	3.5	19.3	7.7	0.6
	CF	-2.2	3.0	10.9	-2.4	4.8	-3.5	7.8	0.0	1.2
	SF	3.6	2.2	7.2	12.7	2.6	-9.6	6.9	3.1	-10.7

FA	-1.9	-2.7	0.5	-5.8	-0.9	5.8	-15.7	-4.2	1.6
NV	4.3	1.3	-5.1	1.6	2.6	-1.8	-7.8	-3.1	4.8
PA	-5.1	-3.8	-1.4	-7.9	-3.3	7.9	-21.6	-5.8	-2.4
DY	-1.4	-0.5	-4.3	-4.8	-3.7	-3.5	-19.6	1.8	0.0
SL	-13.0	-8.7	-13.6	-9.5	-14.1	0.0	-15.7	-6.8	0.0
AP	-1.4	2.2	0.0	0.0	-1.5	12.3	-7.8	-2.4	-2.4
CO	-5.8	3.8	4.3	-1.7	2.2	5.3	7.8	0.6	2.4
DI	10.1	2.2	5.8	7.9	3.7	17.5	-3.9	7.4	7.1
FI	-3.2	-7.3	-5.8	-14.3	-6.8	-3.5	-5.9	-8.3	-2.4
Global Health									
N of patients	22–23	53–57	30–31	20–22	41–42	25–26	18	37–39	25–26
QL	7.6	3.8	-8.1	6.4	3.8	-6.7	13.4	1.3	-4.2
PF	8.0	1.8	-4.1	6.9	-0.4	-1.8	8.1	0.2	1.2
RF	5.1	3.5	-13.4	6.8	-3.2	-8.3	13.0	-6.0	-6.4
EF	6.5	12.1	7.0	17.3	11.2	4.8	13.4	7.9	4.6
CF	1.4	4.1	4.3	-3.0	3.2	3.2	1.9	5.1	-2.6
SF	15.9	5.3	-2.7	2.4	1.6	5.1	11.1	3.8	-7.1
FA	-7.7	-3.5	5.7	-3.3	-2.2	4.7	-9.9	-1.6	-4.3
NV	-5.8	1.5	8.6	0.8	-0.8	3.2	-9.3	-1.3	1.9
PA	-11.6	-3.2	2.7	-8.3	-6.0	5.8	-19.4	-5.1	-7.1
DY	-14.5	-1.2	2.2	0.0	-3.2	-3.8	-5.6	-2.6	0.0
SL	0.0	-12.5	-8.9	-13.6	-6.3	-4.0	-20.4	0.0	-2.7
AP	-10.1	-0.6	17.2	-4.5	0.8	12.8	-5.6	-3.4	5.1
CO	0.0	1.8	4.3	-3.0	4.9	11.5	-3.7	0.9	-2.6
DI	1.4	5.3	6.5	14.3	3.2	5.1	-1.9	2.6	6.4
FI	-15.2	-4.4	-10.8	-6.7	-5.7	-11.5	-14.8	-6.3	-2.6
Change in Performance Status									
N of patients	7	103–104	14–17	6	96–98	14–15	7	79–81	20

QL	31.0	1.7	8.8	29.2	3.3	0.0	23.8	4.5	-4.6
PF	12.4	1.2	1.5	-4.4	2.3	-2.7	8.6	3.3	-6.0
RF	-2.4	-1.1	-3.9	-2.8	-0.9	-2.2	11.9	2.7	-11.7
EF	19.0	8.8	15.7	19.4	11.4	15.0	6.0	9.1	3.5
CF	0.0	3.7	2.9	8.3	3.1	0.0	-2.4	2.7	-5.8
SF	11.9	3.2	12.7	8.3	4.6	-1.1	0.0	4.4	-7.5
FA	-9.5	-1.6	0.0	5.6	-3.5	2.2	-11.1	-4.4	-0.6
NV	0.0	3.9	-12.7	8.3	-0.7	0.0	9.5	-3.3	-0.8
PA	-23.8	-5.3	1.0	-2.8	-7.8	2.2	-2.4	-12.3	-5.0
DY	-19.0	-1.0	-5.9	5.6	-6.1	0.0	0.0	-2.1	-5.0
SL	-9.5	-9.2	-13.7	-16.7	-8.5	-11.9	-9.5	-7.5	5.0
AP	-19.0	3.6	-5.9	-5.6	1.7	-8.9	-14.3	-1.2	-3.3
CO	4.8	2.2	2.0	0.0	3.1	23.8	-23.8	0.4	20.0
DI	0.0	6.7	3.9	5.6	5.8	4.4	23.8	4.1	0.0
FI	4.8	-5.5	-4.8	-5.6	-8.0	-4.4	14.3	-6.3	-6.7

Legend for Supplementary Table 1:

Because the number of patients varied by QOL scale and anchor, we present n as a range for all scales and anchors.

Imp, minimally improved; Det, minimally deteriorated; QL, global quality of life; PF, physical function; RF, role function; EF, emotional function; CF, cognitive function; SF, social function; FA, fatigue; NV, nausea and vomiting; PA, pain; DY, dyspnea; SL, insomnia; AP, appetite loss; CO, constipation; DI, diarrhea; FI, financial problems

Supplementary Table 2. Correlation coefficients of absolute, change, and baseline scores with anchors

Months	Anchor	Scale	n	Absolute score				Change score				Baseline score			
				Pe	Sp	Pc	Ps	Pe	Sp	Pc	Ps	Pe	Sp	Pc	Ps
2	Function														
		QL	130	-0.32	-0.33	-0.38	-0.37	-0.37	-0.39	-0.39	-0.38	0.13	0.08	0.09	0.10
		PF	129	-0.16	-0.16	-0.19	-0.21	-0.30	-0.40	-0.38	-0.33	0.08	0.13	0.10	0.07
		RF	129	-0.11	-0.13	-0.17	-0.17	-0.27	-0.25	-0.31	-0.31	0.12	0.06	0.07	0.11
		SF	130	-0.20	-0.16	-0.26	-0.27	-0.28	-0.24	-0.31	-0.29	0.10	0.02	0.04	0.06
		FA	129	0.24	0.26	0.30	0.31	0.33	0.30	0.34	0.34	-0.04	0.05	0.02	0.00
		DY	128	0.11	0.13	0.16	0.15	0.31	0.29	0.35	0.33	-0.17	-0.12	-0.17	-0.16
		AP	128	0.35	0.33	0.43	0.41	0.34	0.37	0.39	0.37	-0.08	-0.07	-0.08	-0.06
	Concern														
		QL	131	-0.30	-0.27	-0.32	-0.32	-0.31	-0.32	-0.33	-0.32	0.22	0.14	0.20	0.21
	Global Health														
		QL	131	-0.32	-0.33	-0.34	-0.34	-0.45	-0.40	-0.46	-0.46	0.01	-0.01	-0.01	0.00
		PF	130	-0.07	-0.12	-0.11	-0.11	-0.32	-0.39	-0.38	-0.34	0.03	0.10	0.04	0.02
		RF	130	-0.03	-0.10	-0.08	-0.07	-0.26	-0.25	-0.29	-0.30	0.04	0.09	0.05	0.03
		SF	131	-0.15	-0.14	-0.18	-0.20	-0.32	-0.33	-0.37	-0.36	0.00	0.02	0.00	-0.01
		FA	130	0.11	0.19	0.18	0.16	0.31	0.34	0.37	0.34	0.01	0.03	0.04	0.03
		NV	130	0.11	0.13	0.14	0.12	0.24	0.28	0.31	0.26	-0.04	-0.07	-0.08	-0.04
		PA	131	0.05	0.10	0.09	0.08	0.35	0.30	0.35	0.35	-0.10	-0.15	-0.13	-0.10
		DY	129	0.00	0.03	0.03	0.03	0.30	0.25	0.33	0.33	-0.05	-0.04	-0.04	-0.04
		AP	129	0.18	0.22	0.23	0.21	0.31	0.37	0.36	0.34	-0.04	-0.05	-0.04	-0.03
4	Function														
		QL	121	-0.49	-0.45	-0.56	-0.53	-0.39	-0.36	-0.40	-0.40	-0.10	-0.10	-0.14	-0.13
		PF	121	-0.45	-0.31	-0.44	-0.50	-0.37	-0.38	-0.42	-0.41	-0.15	-0.07	-0.13	-0.15
		EF	121	-0.35	-0.29	-0.36	-0.39	-0.32	-0.31	-0.34	-0.32	0.02	0.03	0.01	0.00
		SF	120	-0.50	-0.43	-0.55	-0.55	-0.32	-0.30	-0.36	-0.34	-0.13	-0.15	-0.17	-0.15

		FA	121	0.44	0.37	0.48	0.50	0.46	0.44	0.49	0.48	0.01	0.03	0.06	0.04
		PA	121	0.32	0.24	0.34	0.35	0.31	0.31	0.34	0.32	0.02	-0.06	-0.02	0.04
	Concern														
		QL	120	-0.48	-0.45	-0.52	-0.51	-0.35	-0.34	-0.36	-0.36	-0.14	-0.12	-0.16	-0.15
		SF	120	-0.53	-0.48	-0.58	-0.57	-0.35	-0.35	-0.40	-0.37	-0.12	-0.10	-0.14	-0.13
		PA	120	0.39	0.33	0.41	0.42	0.27	0.31	0.29	0.27	0.12	-0.03	0.06	0.13
	Global Health														
		QL	120	-0.49	-0.45	-0.53	-0.52	-0.31	-0.31	-0.33	-0.32	-0.19	-0.16	-0.21	-0.20
		PF	120	-0.32	-0.20	-0.31	-0.35	-0.29	-0.29	-0.31	-0.29	-0.10	-0.01	-0.11	-0.10
		PA	120	0.25	0.18	0.25	0.27	0.28	0.31	0.31	0.29	-0.01	-0.12	-0.06	-0.02
	Change in Performance Status														
		QL	121	-0.23	-0.17	-0.26	-0.25	-0.31	-0.27	-0.38	-0.36	0.06	0.07	0.10	0.10
6	Function														
		QL	107	-0.38	-0.38	-0.48	-0.46	-0.32	-0.23	-0.32	-0.36	-0.01	-0.03	-0.03	-0.03
		PF	107	-0.31	-0.22	-0.34	-0.40	-0.39	-0.34	-0.42	-0.44	0.05	0.06	0.03	0.02
		RF	107	-0.35	-0.31	-0.46	-0.45	-0.37	-0.38	-0.44	-0.41	0.03	0.06	0.02	-0.02
		EF	107	-0.23	-0.12	-0.24	-0.30	-0.30	-0.22	-0.29	-0.34	0.12	0.13	0.11	0.10
		FA	107	0.29	0.26	0.37	0.39	0.30	0.26	0.33	0.34	0.01	0.03	0.05	0.06
		SL	106	0.23	0.19	0.32	0.33	0.32	0.34	0.41	0.36	-0.12	-0.08	-0.07	-0.08
	Concern														
		QL	111	-0.41	-0.39	-0.44	-0.44	-0.28	-0.25	-0.29	-0.30	-0.08	-0.08	-0.08	-0.08
		PF	111	-0.39	-0.26	-0.37	-0.41	-0.30	-0.30	-0.32	-0.34	-0.11	-0.01	-0.07	-0.10
		RF	111	-0.41	-0.35	-0.45	-0.45	-0.29	-0.35	-0.36	-0.34	-0.09	0.02	-0.04	-0.08
		EF	111	-0.40	-0.26	-0.38	-0.42	-0.43	-0.39	-0.46	-0.46	0.11	0.11	0.11	0.13
		CF	111	-0.22	-0.16	-0.21	-0.23	-0.20	-0.14	-0.21	-0.21	-0.01	0.03	0.01	0.00
		SF	110	-0.50	-0.41	-0.57	-0.53	-0.32	-0.25	-0.33	-0.34	-0.12	-0.12	-0.13	-0.12
	Global Health														
		QL	111	-0.48	-0.47	-0.55	-0.54	-0.42	-0.40	-0.44	-0.44	0.01	0.00	-0.01	-0.01
		PF	111	-0.32	-0.18	-0.32	-0.40	-0.37	-0.31	-0.36	-0.41	0.02	0.09	0.01	-0.01

RF	111	-0.39	-0.33	-0.45	-0.46	-0.42	-0.41	-0.45	-0.44	0.05	0.13	0.08	0.01
EF	111	-0.27	-0.16	-0.28	-0.32	-0.40	-0.35	-0.42	-0.44	0.19	0.16	0.17	0.19
SF	110	-0.39	-0.33	-0.46	-0.46	-0.33	-0.29	-0.35	-0.36	-0.02	-0.01	-0.04	-0.03
FA	111	0.30	0.24	0.33	0.36	0.30	0.24	0.30	0.33	0.02	0.03	0.02	0.05
PA	110	0.40	0.27	0.42	0.47	0.35	0.33	0.37	0.36	-0.03	-0.10	-0.06	-0.01
SL	110	0.23	0.15	0.25	0.26	0.29	0.29	0.33	0.31	-0.09	-0.11	-0.09	-0.08
AP	111	0.35	0.29	0.41	0.40	0.31	0.30	0.34	0.33	0.02	0.03	0.04	0.04
Change in Performance Status													
PF	111	-0.15	-0.11	-0.13	-0.14	-0.30	-0.25	-0.31	-0.31	0.12	0.15	0.18	0.16
RF	111	-0.23	-0.20	-0.23	-0.24	-0.25	-0.25	-0.30	-0.29	0.03	0.04	0.06	0.05
CO	111	0.11	0.11	0.16	0.15	0.26	0.30	0.38	0.34	-0.21	-0.23	-0.34	-0.29
DI	111	-0.20	-0.21	-0.33	-0.26	-0.21	-0.21	-0.30	-0.27	0.04	0.02	0.08	0.06

Legend for Supplementary Table 2:

Pe, Pearson’s correlation coefficient; Sp, Spearman’s rank correlation coefficient; Pc, Polychoric correlation coefficient; Ps, Polyserial correlation coefficient; QL, global quality of life; PF, physical function; RF, role function; EF, emotional function; SF, social function; FA, fatigue; NV, nausea and vomiting; PA, pain; DY, dyspnea; SL, insomnia; AP, appetite loss; CO, constipation; DI, diarrhea

Supplementary Table 3. Estimated differences, p-values, and effect sizes in each pair of anchor and scale

			Improvement			Deterioration		
Months	Anchor	Scale	Estimate (95% CI)	P-value	Effect size	Estimate (95% CI)	P-value	Effect size
2	Function							
		QL	No MID			-5.49 (-17.7, 6.74)	0.375	-0.22
		PF	7.37 (-1.76, 16.50)	0.112	0.31	**-8.27 (-15.1, -1.48)**	**0.018**	**-0.35**
		RF	6.94 (-7.03, 20.92)	0.326	0.23	-10.3 (-20.6, 0.13)	0.053	-0.34
		SF	No MID			No MID		
		FA	No MID			6.07 (-3.36, 15.50)	0.204	0.22
		DY	No MID			8.58 (-2.77, 19.92)	0.137	0.28
		AP	-10.1 (-32.9, 12.74)	0.383	-0.31	No MID		
	Concern							
		QL	7.58 (-5.72, 20.88)	0.261	0.30	-5.46 (-18.8, 7.83)	0.417	-0.21
	Global Health							
		QL	6.23 (-1.09, 13.55)	0.094	0.26	**-11.9 (-23.2, -0.58)**	**0.040**	**-0.47**
		PF	No MID			-5.90 (-12.5, 0.71)	0.080	-0.25
		RF	No MID			**-16.9 (-26.8, -7.09)**	**<.001**	**-0.57**
		SF	10.68 (-1.06, 22.42)	0.074	0.38	-5.15 (-14.6, 4.31)	0.283	-0.23
		FA	-7.26 (-18.8, 4.32)	0.217	-0.33	No MID		
		NV	-8.38 (-17.8, 1.04)	0.081	-0.32	-7.95 (-18.6, 2.65)	0.140	-0.29
		PA	**-13.3 (-25.1, -1.52)**	**0.027**	**-0.43**	**9.24 (0.04, 18.45)**	**0.049**	**0.34**
		DY	No MID			7.14 (-3.32, 17.60)	0.179	0.32
		AP	-9.55 (-27.6, 8.46)	0.296	-0.30	5.90 (-2.61, 14.42)	0.172	0.22
4	Function							
		QL	7.08 (-6.72, 20.87)	0.311	0.28	No MID		
		PF	7.78 (-0.91, 16.47)	0.079	0.33	-6.84 (-14.0, 0.27)	0.059	-0.29
		EF	No MID			No MID		
		SF	6.77 (-7.66, 21.20)	0.353	0.24	No MID		

		FA	-6.84 (-18.9, 5.23)	0.263	-0.25	8.08 (-1.80, 17.96)	0.108	0.29
		PA	No MID			No MID		
	Concern							
		QL	8.17 (-3.51, 19.86)	0.168	0.32	No MID		
		CF	No MID			No MID		
		SF	10.11 (-1.52, 21.73)	0.088	0.36	**-12.2 (-24.3, -0.21)**	**0.046**	**-0.44**
		PA	No MID			11.23 (-0.50, 22.96)	0.060	0.43
	Global Health							
		QL	No MID			-10.5 (-21.6, 0.64)	0.064	-0.41
		PF	7.29 (-0.31, 14.89)	0.060	0.31	No MID		
		PA	No MID			**11.72 (1.50, 21.94)**	**0.025**	**0.44**
	Change in Performance Status							
		QL	No MID			No MID		
6	Function							
		QL	No MID			**-18.5 (-33.9, -3.21)**	**0.019**	**-0.73**
		PF	No MID			**-13.0 (-21.8, -4.19)**	**0.004**	**-0.55**
		RF	No MID			**-20.2 (-34.2, -6.10)**	**0.006**	**-0.67**
		EF	No MID			-10.7 (-22.3, 0.86)	0.069	-0.49
		FA	No MID			11.91 (-0.45, 24.27)	0.059	0.43
		SL	No MID			**23.25 (6.30, 40.20)**	**0.008**	**0.77**
	Concern							
		QL	11.19 (-4.12, 26.49)	0.150	0.44	-8.98 (-25.5, 7.53)	0.282	-0.35
		PF	No MID			-9.11 (-19.3, 1.03)	0.078	-0.39
		RF	**21.12 (6.88, 35.37)**	**0.004**	**0.71**	-15.2 (-30.5, 0.21)	0.053	-0.51
		EF	**11.55 (0.39, 22.71)**	**0.043**	**0.53**	-7.13 (-19.2, 4.91)	0.242	-0.33
		SF	No MID			**-13.8 (-27.3, -0.29)**	**0.045**	**-0.50**
	Global Health							
		QL	12.14 (-2.03, 26.32)	0.092	0.48	-5.45 (-18.0, 7.15)	0.392	-0.21
		PF	7.98 (-0.18, 16.14)	0.055	0.34	No MID		

		RF	18.95 (5.99, 31.90)	0.005	0.63	No MID		
		EF	5.52 (-6.11, 17.15)	0.348	0.25	No MID		
		SF	7.26 (-5.29, 19.82)	0.253	0.26	-10.9 (-22.1, 0.26)	0.055	-0.39
		FA	-8.31 (-19.7, 3.12)	0.152	-0.30	No MID		
		PA	**-14.3 (-26.5, -2.12)**	**0.022**	**-0.54**	No MID		
		SL	**-20.4 (-36.6, -4.15)**	**0.014**	**-0.67**	No MID		
		AP	No MID			8.55 (-7.55, 24.64)	0.294	0.27
	Change in Performance Status							
		PF	5.32 (-8.77, 19.41)	0.456	0.27	**-9.25 (-18.2, -0.32)**	**0.042**	**-0.46**
		RF	9.23 (-12.1, 30.51)	0.392	0.35	**-14.3 (-27.8, -0.85)**	**0.037**	**-0.55**
		CO	No MID			No MID		
		DI	No MID			-4.12 (-15.3, 7.07)	0.467	-0.21
All	Function							
		QL	**11.22 (3.69, 18.75)**	**0.004**	**0.44**	-5.76 (-13.8, 2.32)	0.162	-0.23
		PF	4.83 (-0.37, 10.03)	0.069	0.21	No MID		
		RF	No MID			**-8.38 (-16.2, -0.61)**	**0.034**	**-0.28**
	Concern							
		QL	No MID			**-7.72 (-14.5, -0.93)**	**0.026**	**-0.30**
		EF	No MID			**-5.25 (-10.1, -0.36)**	**0.035**	**-0.24**
	Global Health							
		QL	No MID			**-10.4 (-16.7, -4.00)**	**0.001**	**-0.41**
		PF	**4.84 (0.77, 8.91)**	**0.020**	**0.21**	No MID		
		RF	**6.77 (1.25, 12.29)**	**0.016**	**0.23**	**-6.30 (-12.2, -0.36)**	**0.038**	**-0.21**
		SF	No MID			**-7.32 (-12.5, -2.12)**	**0.006**	**-0.26**
		FA	No MID			**6.59 (2.30, 10.88)**	**0.003**	**0.24**
		PA	**-6.69 (-12.4, -0.99)**	**0.021**	**-0.25**	No MID		

Legend for Supplementary Table 3:

The mean change method was applied for months 2, 4, and 6; the GEE method was applied for overall timepoints. Estimates with P < 0.05 are shown in **bold**.

QL, global quality of life; PF, physical function; RF, role function; EF, emotional function; SF, social function; FA, fatigue; NV, nausea and vomiting; PA, pain; DY, dyspnea; SL, insomnia; AP, appetite loss; CO, constipation; DI, diarrhea
